# Immunomodulatory effects of *Ziziphora tenuior* L. extract on the dendritic cells

**DOI:** 10.1186/s40199-014-0063-8

**Published:** 2014-09-17

**Authors:** Abbas Azadmehr, Robabeh latifi, Sahar Mosalla, Reza Hajiaghaee, Mojtaba Shahnazi

**Affiliations:** Immunology Department, Qazvin University of Medical Sciences, Qazvin, Iran; Parasitology Department, Qazvin University of Medical Sciences, Qazvin, Iran; Pharmacognosy and Pharmaceutics department of Medicinal Plants Research Center, Institute of Medicinal Plants, (ACECR), Karaj, Iran

**Keywords:** *Ziziphora tenuior* L, Immunomodulation, Dendritic cells, T cell responses, Cytokine

## Abstract

**Background:**

*Ziziphora tenuior* L. (Kakuti in Persian) is used in traditional medicine for treatment of gastrointestinal disorders as carminative and analgesic plant. The other usages of this plant are included treatment of diarrhea and nausea. Therefore in the present study we evaluated the immunomodulatory effects of the ethanolic extract of this plant on the dendritic cells (DCs).

**Results:**

*Ziziphora tenuior* L. extract significantly (p = 0.002) increased the level of surface expression of CD40 as an important co-stimulatory marker on DCs compared to the control. However this extract did not change CD86 and MHC-II molecules, so it could promote DCs phenotypic maturation. Treatment of DCs with the extract resulted in slightly increased of the production of (IL-12); however, this change was not significant. In addition, the ability of treated DCs to stimulate allogenic T cells proliferation and cytokines secretion was examined in the co-cuture of these cells with T cells in mixed lymphocyte reaction (MLR). *Z. tenuior* L. at the 100 μg/ml concentration inhibited the proliferation of allogenic T cells and also significantly (P < 0.001) increased the level of IL-10. Moreover, the extract at 10–100 μg/ml concentration caused slightly increase in IFN-γ production and decreased IL-4 cytokines but these changes were not significant.

**Conclusions:**

These findings indicated that *Z. tenuior* L. extract can modulate immune response by induction of CD40 expression on DCs and cytokine production; whereas it can inhibit T cell stimulating activity of DCs in high concentration. These findings possibly in part explain the traditional use of this plant in treatment of immune-mediated disorders. However future studies are needed.

## Introduction

Medicinal plants are used to treat immune disorders, such as inflammatory and autoimmune diseases in the worldwide. Previous studies showed that some herbal medicine have immunomodulatory effects [1,2]. *Ziziphora* genus (Lamiacaea) is included of four species namely *Z. tenuior* L., *Z.capitata* L., *Z.clinopodioides* and *Z. Persica Bunge* [3]. *Ziziphora* or Kakuti in Iranian traditional medicine is recognized to dried aerial parts of *Z. tenuior* L. which contains at least 1.2% (volum/weight) of the essential oil [4]. This medicinal plant distributed widely in Iran, Pakistan, Turkmenistan, Afghanistan, Armenia, Anatolia, Syria, Transcaucasia and Central Asia [3]. In traditional medicine, *Ziziphora* is used to treat dysentery, fever, uterus infection and also as analgesic plant [5]. In addition, it is used in the treatment of the gastrointestinal disorder as carminative or treat of vomiting or diarrhea [6,7]. Phytochemical analysis indicated that major components of its essential oil are pulegone, isomenthone, thymol, menthone and piperitone [7-10]. Previous studies showed that these compounds are suggested to be responsible for the mentiond medicinal properties. It has been shown that, *Ziziphora clinopoides* methanolic extract inhibited inflammatory mediators in dextran sodium sulfate induced colitis in mice and also protected acetic acid-induced toxic bowel inflammation through reduction of cellular lipid peroxidation and myeloperoxidase activity [11,12]. On the other hand, Dendritic cells are potential antigen presenting cells for naive T cells and act as a link between the acquired and innate immune systems for the initiation of the protective immune response or the induction of immune tolerance [13]. Maturation status, origin and phenotype are affected on the function of these cells [14]. The nature of the cytokines produced by DCs in response to various ligands eventually modulates and determines the type of Th cell response. DCs have the exclusive ability to stimulate naive T cells to either Th1 or Th2 cells and also effectively down-regulate T-cell responses through the generation of T regulatory cells [15,16]. It has been demonstrated that immature DCs can effectively present antigen to naive T cells but because of low expression of co-stimulatory molecules such as CD40, CD86 and MHC II they cannot suitably stimulate immune system, finally T cells activation and proliferation are inhibited [14]. In this regards, maturation of DCs converts them to the cells that can stimulate immune system. So, the use of these cells as therapeutic targets by pharmacological compounds such as medicinal plant is a valuable strategy to modulate immune responses. In the present study we have evaluated the immunomodulatory effects of *Z. tenuior* L. extract on the function and maturation of DCs.

## Materials and methods

### Animals

The male BALB/c and C57BL/6 mice with 6–8 week old were purchased from Razi Institute (Karaj, Iran) and were kept under optimal conditions of hygiene and received standard mouse chow and water *ad libitum*. In this study, all experimental procedures on handling the animals were approved by the ethical committee of Qazvin University of Medical Sciences.

### Preparation of DCs from mice spleen

To isolate DCs from spleen, the mice CD11c^+^ isolation kit (Miltenyi Biotech, Germany), was used as described in previous study [17]. Briefly, the mice spleens were isolated and minced to very small pieces and then suspended in cold (4°C) PBS containing 1.2 mg/ml collagenase D (Roche, Germany).The cell suspension, incubated at 37°C for 40 minutes and was passed through a cell mesh and the collected cells were washed twice. Then, mononuclear cells (MNC) were separated by Nycodenz (Axis-shield, Norway); a material used in density gradient cell separation techniques. According to the manufacturer’s protocol, anti-CD11c magnetic beads (Miltenyi Biotech, Germany) were added to the cells and incubated at 4°C for 30 minutes and the non-bonded antibodies were washed out. Using of the minimacs separator (MS) cell columns into the magnetic field (MS separation unit, Miltenyi Biotech, USA) and washed by 1.5 ml EDTA-containing PBS and then passed through the column to absorb the magnetic bead coated DCs and the unwanted cells were washed out using at least 3 washes of 500 μl EDTA-containing PBS. The isolated DCs were collected by disconnecting the column from the magnetic field and injecting 1 ml PBS-EDTA in it under pressure. Purity of the CD11c dendritic cells was determined above 94% using flow cytometry.

### Plant material and preparation of the ethanolic extract

*Ziziphora tenuior* was purchased from herbal market and authenticated by a botanist. Voucher specimens were preserved in the central herbarium of medicinal plants (ACECR). The aerial parts of plant were air-dried at room temperature. This was ground in to powder. The aerial part of plant (50 g) was extracted using percolation method by ethanol (80%) at room temperature. Solvent was completely removed by drying under reduced pressure at 40°C in a rotary evaporator. The samples were stored at 4°C until use (3 g, 6% yield) [18].

### Effect of extract on DCs viability

To determine, the effect of cytotoxic concentration of the extract on DCs, 3-(4, 5-dimethylthiazol-2-yl)-2, 5-diphenyltetrazolium bromide (MTT) colorimetric assay was used [15]. Briefly, dendritic cells (10^5^ cells/well) were treated with *Z. tenuior* L. extract at 10 to 200 μg/ml concentrations in the cell culture plates for 24 h, and then 10 μl MTT (5 mg/ml, Sigma) was added to each well and cells were incubated for an additional 4 h at 37°C. Dimethyl sulphoxide (DMSO 0.1%) as vehicle and lipopolysaccharides (LPS 1 μg/ml) as DC maturation inducing agent, were used as negative and positive controls, respectively. Finally, the optical density (OD) of each well was measured at 570 with reference at 630 nm on an enzyme-linked immuosorbent assay (ELISA) plate reader and the viability was determined as the follows: (OD of extract-treated cells/OD of DMSO-treated cells) × 100.

### Analysis of markers expression of co-stimulatory molecules

In order to evaluate the effect of *Z. tenuior* L. on the expression of co-stimulatory molecules, DCs were treated with the extract for 18 h and analyzed using flow cytometer (FACSCalibur, Beckton Dickinson Biosciences, San Jose, CA). The cells were stained with phycoerithrin (PE)-conjugated anti-CD11c, FITC-conjugated anti-CD86, fluorescence isothiocyanate (FITC)-conjugated anti-CD40, FITC-conjugated anti-MHC II antibody and appropriate conjugated isotypes (Beckton Dickinson (BD) Pharminogen, San Diego, CA). Finally, the percentage of markers expression on extract-treated DCs and DMSO-treated DCs was calculated and the mean florescent intensity (MFI) was analyzed using Win MDI software (Scripps, La Jolla, CA).

### Mixed lymphocyte reaction

To determine the proliferative effect of *Z. tenuior* L. extract-treated DCs on T lymphocytes, allogeneic mixed lymphocyte reaction (MLR) assay was used. Briefly, T cells were purified from lymph nodes of C57BL/6 mice using nylon wool. Then, *Z. tenuior* L -treated DCs from BALB/c mice were inactivated with mitomycin C (0.5 mg/ml) for 20 min, and then cells were washed with phosphate buffered saline (PBS) for three times and resuspended in culture medium containing 10% FCS. Mitomycin-treated DCs (10^4^ cells/well), as stimulator cells, co-cultured with 10^5^ allogenic T cells, as responder cells, in a 96-well culture plate (Nunc, Denmark) in triplicates for 48 h. As negative control, a triplicate wells containing DMSO-treated DCs plus allogenic T cells were used. Finally, T cell proliferation was measured by a 5-Bromo-20-deoxy-uridine (BrdU) cell proliferation assay kit (Roche, Germany) according to the manufacturer’s instructions. The result was concluded as the follows: (OD of extract-treated culture/OD of DMSO-treated culture) × 100.

### Effect of the extract on the cytokines production

In order to evaluate immunomodulatory effect of *Z. tenuior* L. extract on the cytokines production, the enzyme-linked immunosorbent assay (ELISA) method was used. Briefly, the supernatant of extract-treated DCs and MLR cultures were collected and used to measure IFN-γ, IL-4, IL-12 and IL-10 by ELISA kits according to the manufacturer’s protocol (eBioscience, USA), respectively.

### Statistics analysis

In this study, all data were representative of at least three independent experiments performed in triplicate and presented as mean ± standard deviation (SD). Mann–Whitney test and one-way analysis of variance (ANOVA) were used for the evaluation of statistical differences between the results and p vales less than 0.05 were considered significant.

## Results

### Viability of DCs after exposure with *Z. tenuior* L. extract

**Figure 1:**
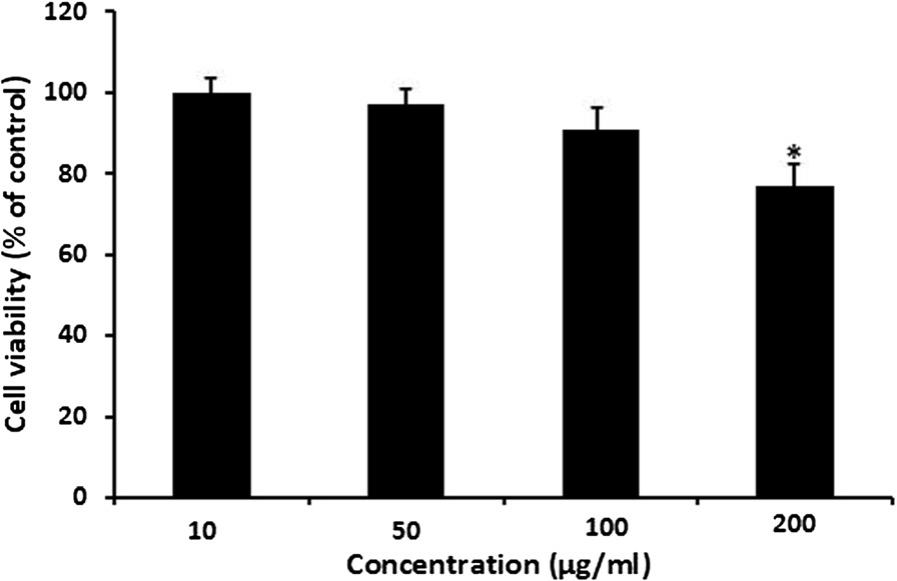
**DCs viability after treatment with** ***Z. tenuior*** **L. extract.** MTT assay was used for determining of DCs viability. These cells were treated with different concentrations of the plant extract for 24 h. DCs treated with DMSO was used as control. The results show mean ± standard deviation of three independent experiments performed in triplicate. The extract at 10 to 100 μg/ml had no significant growth inhibitory effects on DCs. *Significantly (P < .0.05) different was between the extract-treated cells and the control cells.

The DCs viability was determined using MTT assay. For this purpose, these cells were treated with different concentrations of the plant extract for 24 h. Our results indicated that this extract at concentration of 10, 50 and 100 μg/ml had no cytotoxic effect on DCs (Figure 1) however the extract at 200 μg/ml had significantly (P < 0.05) cytotoxic effect on DCs. Therefore these concentrations (10 to 100 μg/ml) were used as safe dose of *Z. tenuior* L. extract for the next experiments on DCs.

### Effect of *Z. tenuior* L. extract on maturation phenotype of DCs

**Figure 2:**
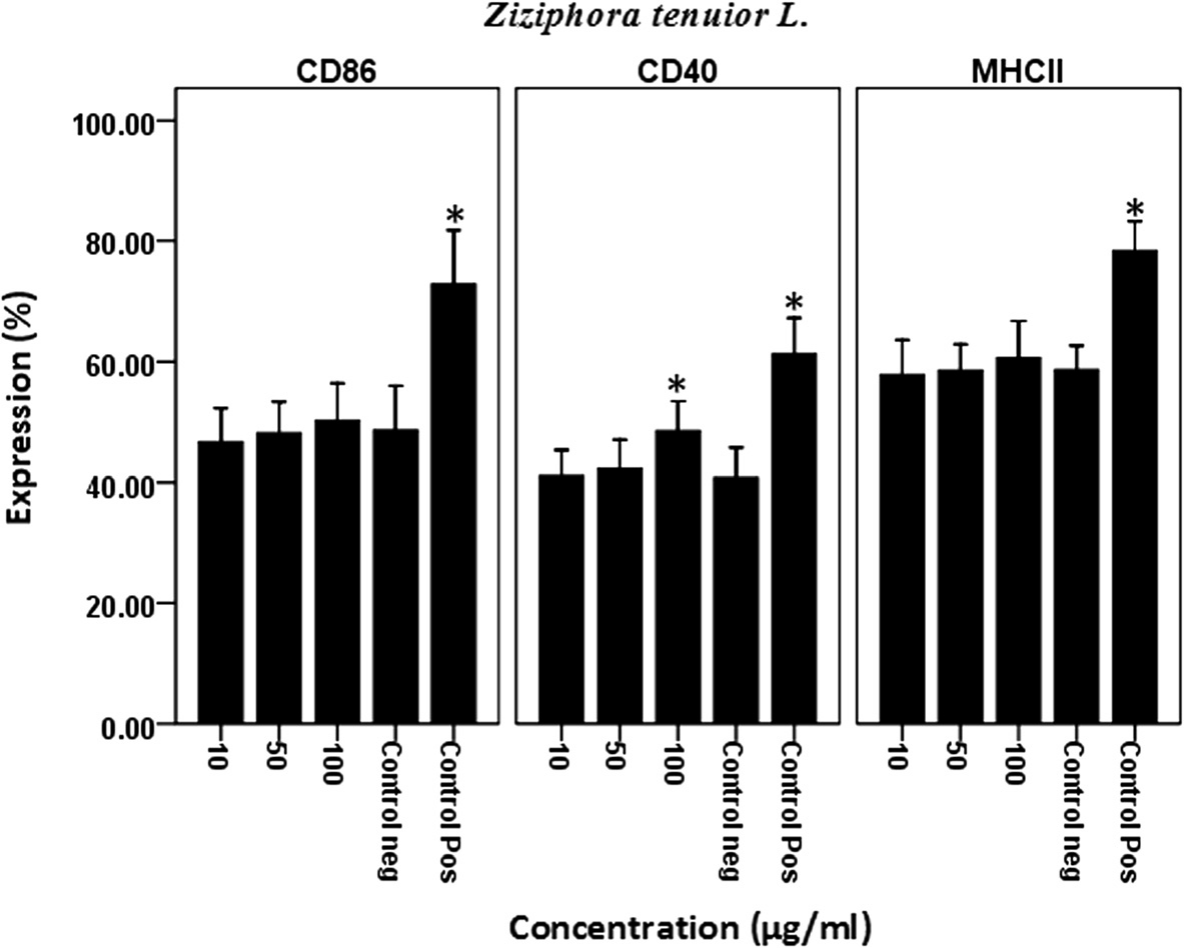
**Effect of** ***Z. tenuior*** **L. extract on maturation phenotype of DCs.** In order to determine the effect of *Z. tenuior* L. extract on maturation phenotype on DCs, these cell were treated with the extract for 18 h and then the expression of CD40, CD86 and MHC II molecules was determined by flow cytomety. DC treated with DMSO as negative control and also lipopolysaccharides (LPS 1 μg/ml) as positive control were considered. The results show mean ± standard deviation of three independent experiments performed in triplicate. *Significantly (P < 0.05) different was between the extract-treated cells and the negative control cells.

We analyzed the percentage expression and fluorescent intensity of CD86, CD40 and MHC II co-stimulatory molecules on *Z. tenuior* L-treated DCs by flow cytometry. Our results showed that the extract modulate the percentage expression and fluorescent intensity of CD86, CD40 and MHC II molecules on DCs. As shown in Figure 2, this extract significantly (P = 0.002) increased the percentage expression of CD40 molecules at 100 μg/ml compared with negative control. Although, *Z. tenuior* L. Extract increased the percentage expression and fluorescent intensity of CD86 and MHC II on DCs but this effect was not significant comparing with control. These finding showed that *Z. tenuior* L. extract can modulate the maturation phenotype markers on DCs.

### Effect of *Z. tenuior* L. extract on allogenic MLR

**Figure 3:**
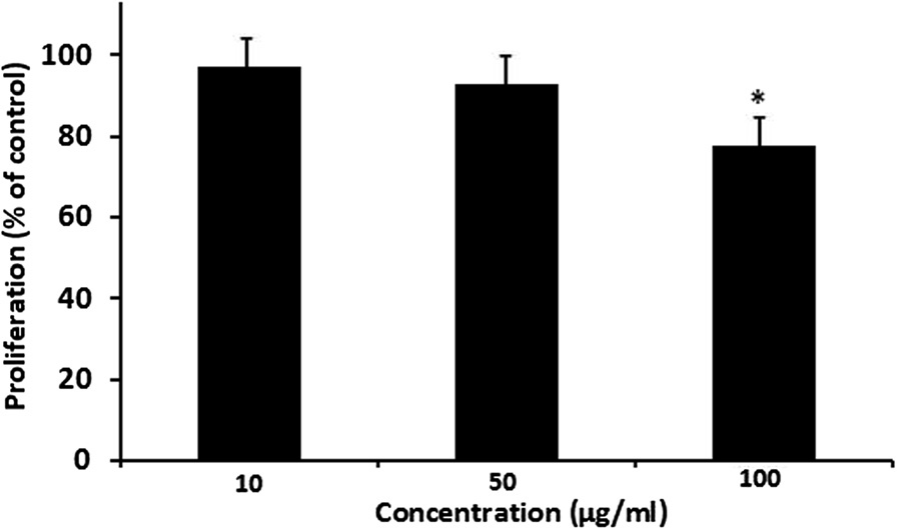
**The effect of** ***Z. tenuior*** **L. extract on allogenic MLR.** In order to find the effects of the *Z. tenuior* L. extract on allogenic immune response, DCs were treated with the extract for 18 h and then co-cultured with allogenic T cells for 48 h. DMSO-treated DCs plus T cells was considered as control. Brdu incorporation assay was used for measuring of cell proliferation. The results show mean ± SD of the cell proliferation in the presence of the extract as compared to the proliferation of controls taken to be 100%. Our results indicated that DCs treated with 100 μg/ml of extract significantly (*P < 0.05) have decreased T cells proliferation.

To determine the effects of the *Z. tenuior* L. extract on allogenic immune response and DCs function, the allogenic T cells from C57BL/6 were co-cultured with DCs isolated from BALB/c mice. For this purpose, the DCs were treated with concentrations of 10 to 100 μg/ml of the extract for 18 h and then cells were co-cultured with allogenic T cells in MLR assay. Finally, the proliferation of T lymphocytes was evaluated using BrdU incorporation assay. As it is shown in Figure 3, the *Z. tenuior* L. extract significantly (p < 0.05) decreased the proliferation of T cells at 100 μg/ml. The results indicated that the proliferation of these cells decreased to 76.8 ± 3.4 percent of control when DCs had been treated with 100 μg/ml of the extract concentration.

### Effect of *Z. tenuior* L. on the production of cytokines

**Figure 4:**
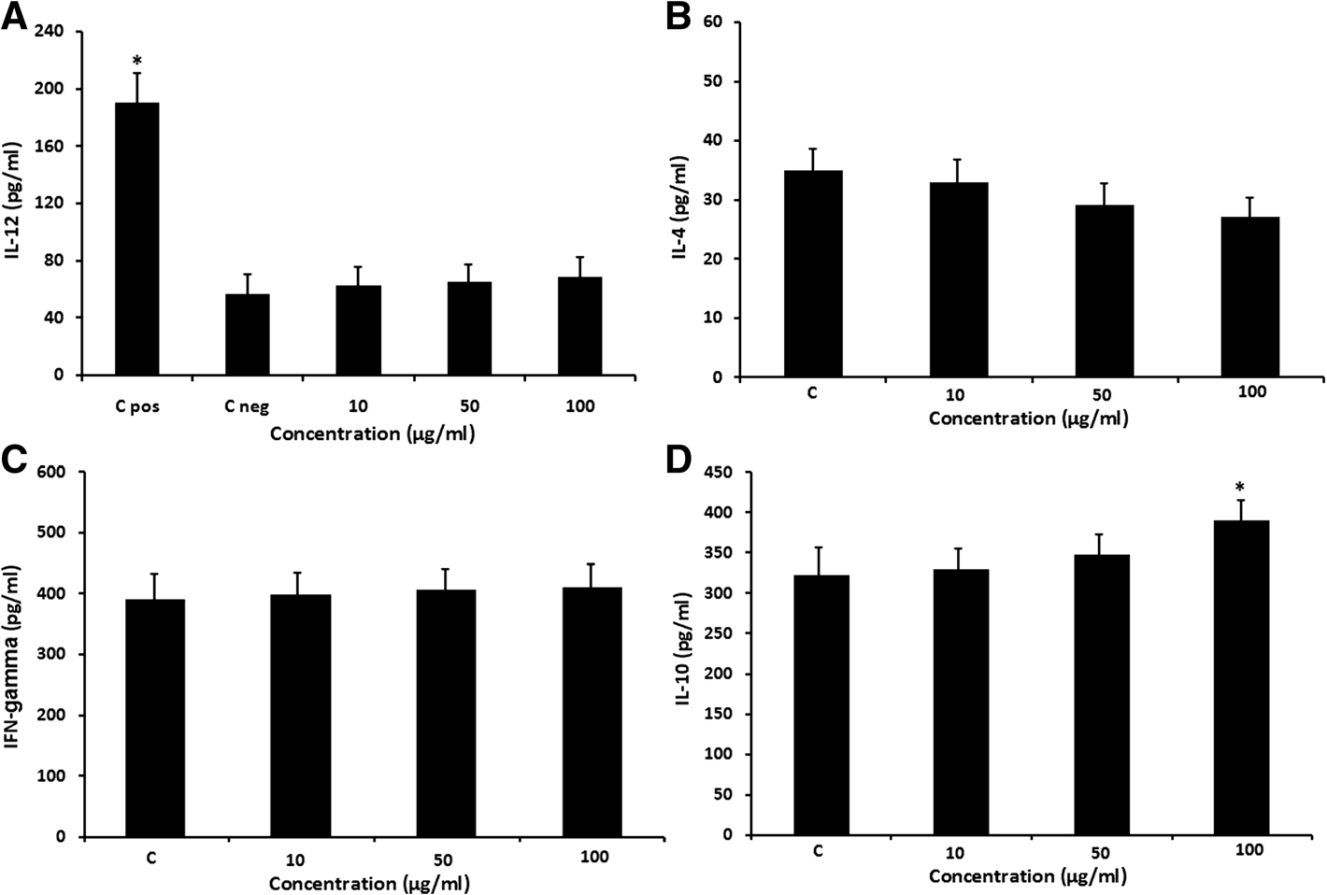
**Effect of** ***Z. tenuior*** **L. extract on cytokine production.** The effect of the *Z. tenuior* L. extract on IL-12 production by DCs after 18 h showed in Figure 4**A**. DMSO treated DCs as control negative (C neg) and LPS as positive control (C pos) were considered. Moreover, the effect of the extract on IFN- γ, IL-4 and IL-10 production in MLR assay showed in Figure 4**B**, **C** and **D**. DMSO-treated DCs plus allogenic T cells as Control considered. *P < 0.05 shows significant difference with the negative control.

The concentration of the IL-12 cytokine in the supernatant of the extract-treated DCs was measured. As shown in Figure 4A, treatment of DCs with this extract at 10 to 100 μg/ml resulted in slightly increase of production of IL-12 by these cells comparing with the negative control. However this change was not significant. In addition, we measured the effect of the extract on IFN- γ, IL-4 and IL-10 production in MLR. We found that, the extract at 10–100 μg/ml concentrations slightly increased production of IFN-γ and decreased IL-4 cytokines but these changes were not significant (Figure 4B, C). However, as it is shown in Figure 4D, our result indicated that the *Z. tenuior* L. extract at 100 μg/ml concentration significantly (p < 0.05) increased IL-10 cytokine production.

## Discussion

In this study, we evaluated the immunomodulatory effects of *Z. tenuior* L. extract on the DCs. Several studies showed that natural agents such as plants can modulate DCs activity [19-21]. Dendritic cells are professional antigen presenting cells for naive T cells and act as a link between the acquired and innate immune systems for the initiation of the protective immune response or the induction of immune tolerance [13]. Therefore, to find out the new DCs immunomodulaters are considered as the important targets for immune response as well as immune regulation. *Ziziphora* is used to treat dysentery, fever, uterus infection and also analgesic plant in traditional medicine [5]. In addition, it is used in the treatment of the gastrointestinal disorder as carminative or treat of vomiting or diarrhea [6,7]. It has been shown that, *Ziziphora clinopoides* methanolic extract inhibited inflammatory mediators in dextran sodium sulfate induced colitis in mice and also protected acetic acid-induced toxic bowel inflammation through reduction of cellular lipid peroxidation and myeloperoxidase activity [11,12].

In the present study, we examined the effect of the *Z. tenuior* L. extract on the phenotypic maturation and function of DCs. The expression of CD86, CD40 and MHC- ІІ molecules are important co-stimulatory and maturation markers on DCs and have critical roles in antigen presentation and T cell activation. Our results showed that the extract modulate the percentage expression and fluorescent intensity of CD86, CD40 and MHC II molecules on DCs. Previous studies indicated that some herbal extracts can modulate immune response by immunomodulatory effect on the CD40 expression on the dendritic cells [22,23]. Our results showed that the extract significantly increased the percentage expression of CD40 molecules at 100 μg/ml compared with control negative. Although, *Z. tenuior* L. extract increased the percentage expression and fluorescent intensity of CD86 and MHC II on DCs but this effect was not significant comparing with control. These finding indicated that *Z. tenuior* L. extract can modulate the maturation phenotype markers on DCs.

In addition, we evaluated the immunomodulatory effect of *Z. tenuior* L. extract on the DCs functions by releasing of cytokines and MLR. IL-12 is a cytokine released by DCs which can induce differentiation of T cells to Th1 and cellular immunity, by contrast IL-10 is a Th1 inhibitory cytokine and also, IL-4 and IFN-γ cytokines are landmark of deviation to Th1 or Th2 response [24]. Our results indicated that the treatment of DCs with this extract at 10 to 100 μg/ml concentration increased production of IL-12 comparing with the negative control however this change was not significant. Also, the extract at 10–100 μg/ml concentrations increased production of IFN-γ and decreased IL-4 cytokines but these changes were not significant. However, our result indicated that the *Z. tenuior* L. extract at 100 μg/ml concentration significantly increased IL-10 cytokine production. We assumed that the increased IL-12 production by DCs using 10–100 μg/ml extract concentration along with production of more IFN-γ and less IL-4 by T cells in MLR suggest the ability of *Z. tenuior* L. extract to deviate the cytokine pattern of T cells toward a Th1 response. However, the extract at 100 μg/ml concentration has increased IL-10 secretion in MLR and also reduced the proliferation of T cells in allogenic response, which indicated the inhibitory effect of the extract at higher concentration on the immune response. Moreover, the difference between the effects of the extract at higher concentrations may be attributed to the presence of various constituents in the extract with different mode of actions. So, to confirm Th1/Th2 polarization in co-culture of T cells with *Z. tenuior* L. treated-DCs, study of the T cell signaling activity of DCs and the expression of T bet and GATA-3 as related transcription factors for Th1 and Th2 differentiation is recommended [25]. Future studies are suggested for identifying the main bioactive compound of this plant and other immunomodulatory mechanisms.

## Conclusions

Our findings indicated that *Z. tenuior* L. extract can modulate immune response by induction of CD40 expression on DCs and cytokine production; whereas it can inhibit T cell stimulating activity of DCs in high concentration. These findings possibly in part explain the traditional use of this plant in treatment of immune-mediated disorders. However future in vitro and in vivo studies are needed.
